# Autoinflammation It Is!

**DOI:** 10.3390/jcm10215157

**Published:** 2021-11-03

**Authors:** Eugen Feist

**Affiliations:** Department of Rheumatology, Helios Clinic Vogelsang-Gommern, Cooperation Partner of the Otto-von-Guericke University, Sophie-von-Boetticher-Straße 1, 39245 Vogelsang-Gommern, Germany; Eugen.Feist@helios-gesundheit.de

In recent years, we have entered a new era full of insights into exciting pathways and improved management of a distinct class of inflammatory conditions. Under the umbrella of auto-inflammation, several so far seemingly unconnected diseases have been summarized and separated from autoimmune conditions in particular. Initially, the striking difference became clear for classical monogenetic periodic fever syndromes, where the innate immune system plays a dominant role via the impact of a specific cytokine signature. 

In this Special Issue of the *Journal of Clinical Medicine*, we find a diverse spectrum of excellent contributions to this topic. Articles on the current knowledge of cryopyrin-associated periodic syndromes [1] and proteasome-associated autoinflammatory syndromes [2] provide us with valuable advice for diagnosis and treatment of these rare conditions in clinical practice. 

The field of auto-inflammation has further extended to more complex polygenetic disorders. In this context, two diseases, namely adult-onset Still’s disease [3] and gouty arthritis [4], are focused on in this Special Issue. Both contributions show that in these clinically and mechanistically diverse diseases, the main symptoms are caused by a very similar cytokine signature referring to interleukin 1 as the main driver. This knowledge has also paved the way for new targeted and highly effective therapies. 

This Special Issue on auto-inflammation also contains relevant contribution that have been rarely addressed in such detail elsewhere. To highlight just a few points, one can read about the current options of imaging in auto-inflammation [5] with special attention to IgG4 related diseases [6] or about the issue of dysphagia in myositis [7]. Furthermore, this Special Issue also contains valuable original work, such as studies providing basic data on a novel variant of TNF receptor-associated periodic syndrome [8], drug hepatotoxicity in the treatment of gouty arthritis [9], the impact of certain anti-rheumatic drugs on DNA repair [10], genetic background in association with response to anti-rheumatic drugs [11], and the impact of IL1-inhibition on the cytokine milieu in adult-onset Still’s disease [12].

Auto-inflammation can also cause life-threatening complications often in association with hyper-inflammation or cytokine storm. Nowadays, everyone is familiar with the issue of severe COVID-19 infections due to an overwhelming and disturbed cytokine signaling. Of note, during this pandemic disaster, special attention has been attributed to the group of patients receiving immunosuppressive therapy, which typically includes those with auto-immune and -inflammatory diseases. In this Special Issue, we are able to publish one of the earliest observations with respect to rare auto-inflammatory diseases. The risk for severe acute COVID-19 disease was mild to moderate in these patients [13]. However, even after resolution of infection, there was an impact on disease activity in these cases. With respect to hyper-inflammation, the critical role of interferons has recently been highlighted. In this context, we can also learn a lot from complications in auto-inflammatory diseases, such as macrophage activation syndrome [14].

Research in the field of auto-inflammation remains in its infancy. Currently, we cannot place each manifestation in a distinct category. To solve this problem, the concept of systemic undefined recurrent fevers has recently been introduced [15]. We should be aware that many of these diseases are still under-recognized, which requires our special attention. Overall, this Special Issue on auto-inflammation is of valuable interdisciplinary information. I am very thankful to the authors for their contribution to this constantly growing field and wish you a fruitful reading.
